# Supplementary material for: Comparison of High- and Low-LET Radiation-Induced DNA Double-Strand Break Processing in Living Cells
Source: Int J Mol Sci. 2020 Sep 9;21(18):6602. doi: 10.3390/ijms21186602 (PMC7555951; doi:10.3390/ijms21186602)
Supplement: Supplementary file 1 [file ijms-21-06602-s001.zip › ijms-884282-supplementary final/ijms-884282-supplementary caption.docx]

Supplementary material

Supplemental movie 1.

Representative movie of a segmented U2OS nucleus containing 53BP1-GFP. The cell was irradiated with X-rays (2 Gy) and imaged using confocal microscopy for 17 hours. Each frame represents 20 min.

Supplemental figure S1.

MSD curves of all binned tracks lengths. α-particle irradiated cells are depicted using a red line and X-ray irradiated cells in blue.

Supplemental figure S2.

Overview of foci intensity distribution with 100 min intervals after the start of imaging. Graphs show kernel density estimations with the area under the curve being equal to the area of the histogram. Histogram bars are representative of the absolute values. The 0 AUs are the consequence of the smoothing procedure used for density estimations.

Supplemental figure S3.

Overview of foci size distribution with 100 min intervals after the start of imaging. Graphs show kernel density estimations with the area under the curve being equal to the area of the histogram. Histogram bars are representative of the absolute values. The 0 AUs are the consequence of the smoothing procedure used for density estimations.

Supplemental figure S4

Overview of 53BP1-GFP amount per foci distribution with 100 min intervals after the start of imaging. Graphs show kernel density estimations with the area under the curve being equal to the area of the histogram. Histogram bars are representative of the absolute values. The 0 AUs are the consequence of the smoothing procedure used for density estimations.
