# Supplementary figures and images for: Comparison of High- and Low-LET Radiation-Induced DNA Double-Strand Break Processing in Living Cells
Source: Int J Mol Sci. 2020 Sep 9;21(18):6602. doi: 10.3390/ijms21186602 (PMC7555951; doi:10.3390/ijms21186602)

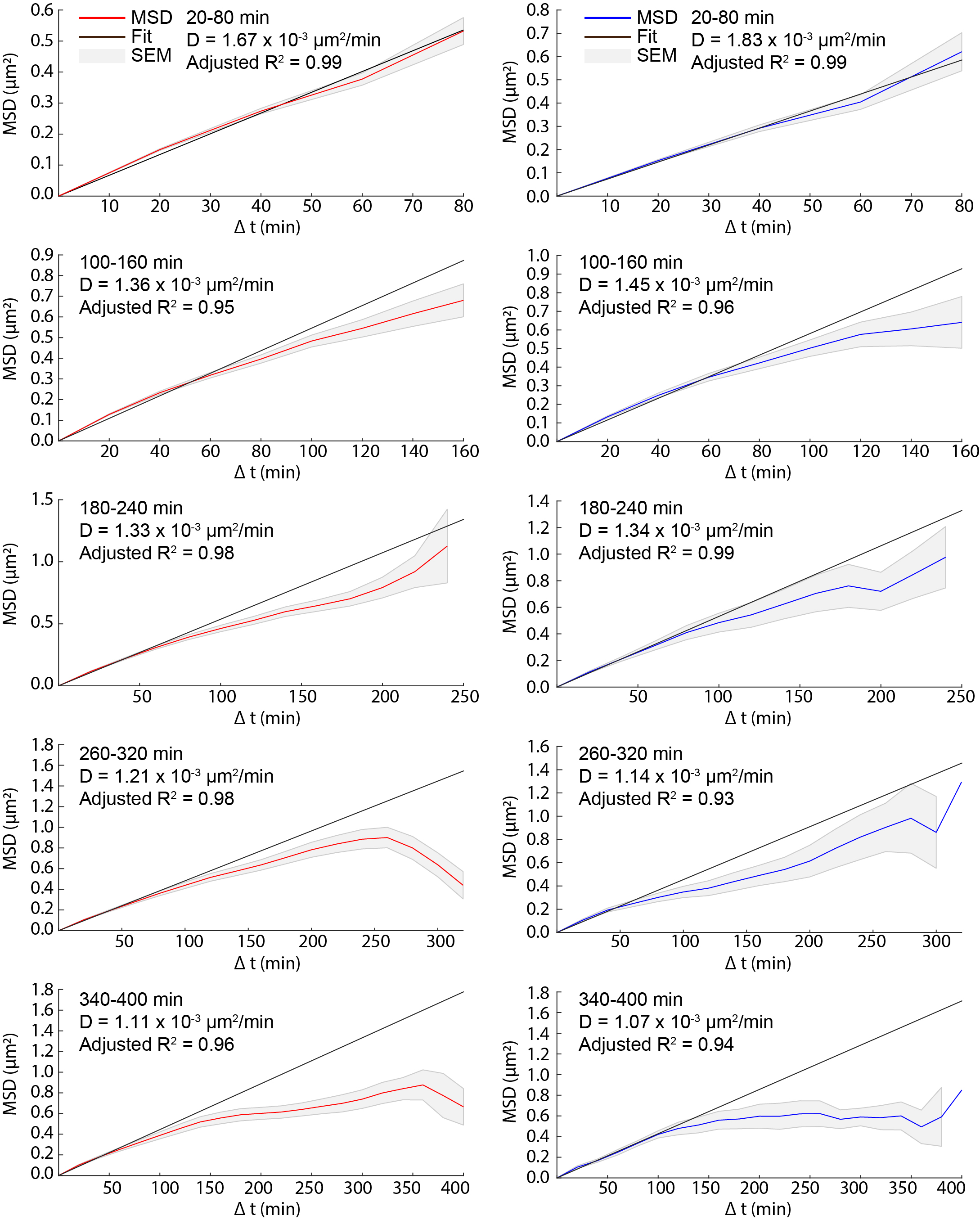

Supplement: Supplementary file 1 [file ijms-21-06602-s001.zip › ijms-884282-supplementary final/Figure S1.jpg]

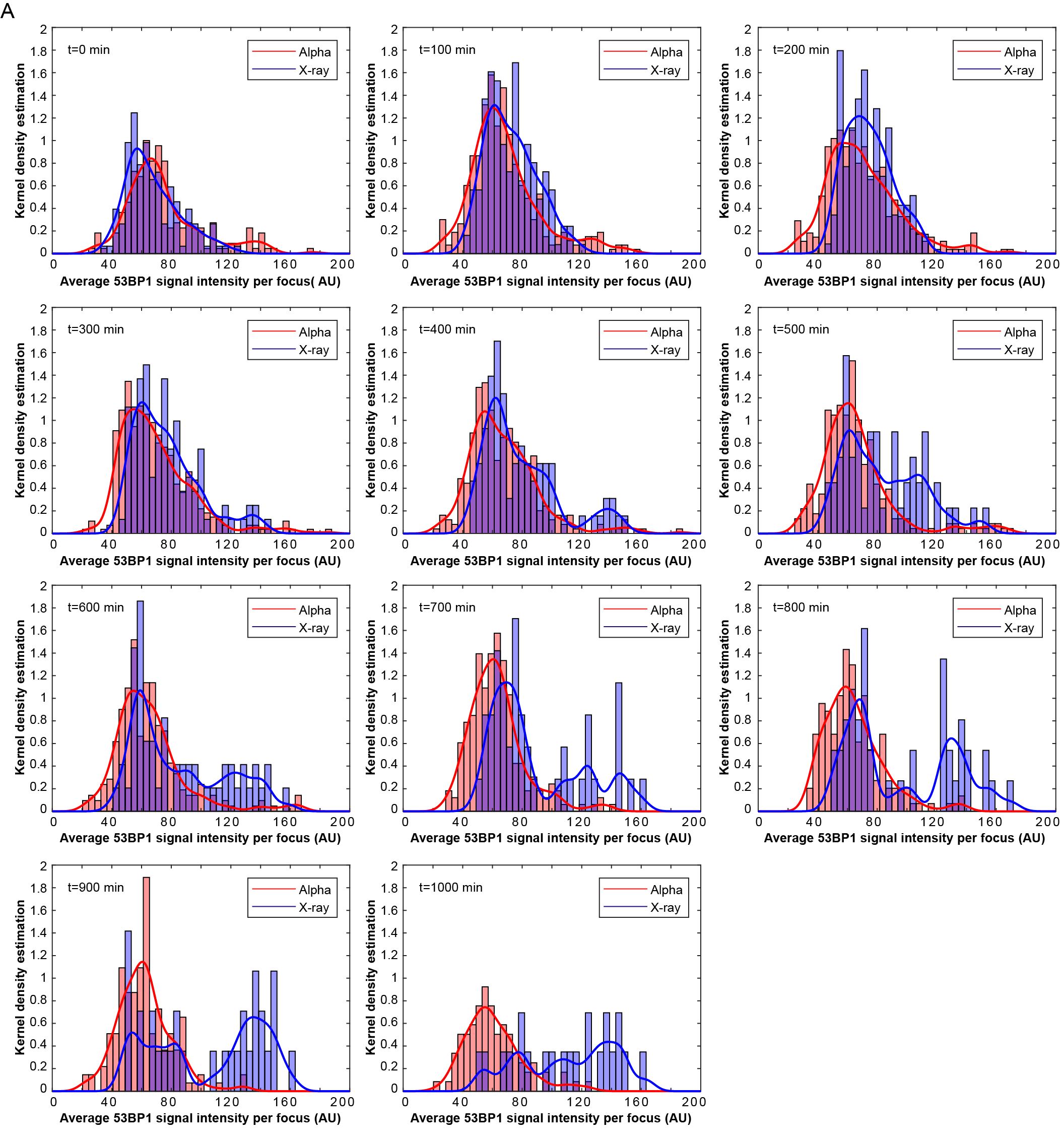

Supplement: Supplementary file 1 [file ijms-21-06602-s001.zip › ijms-884282-supplementary final/Figure S2.jpg]

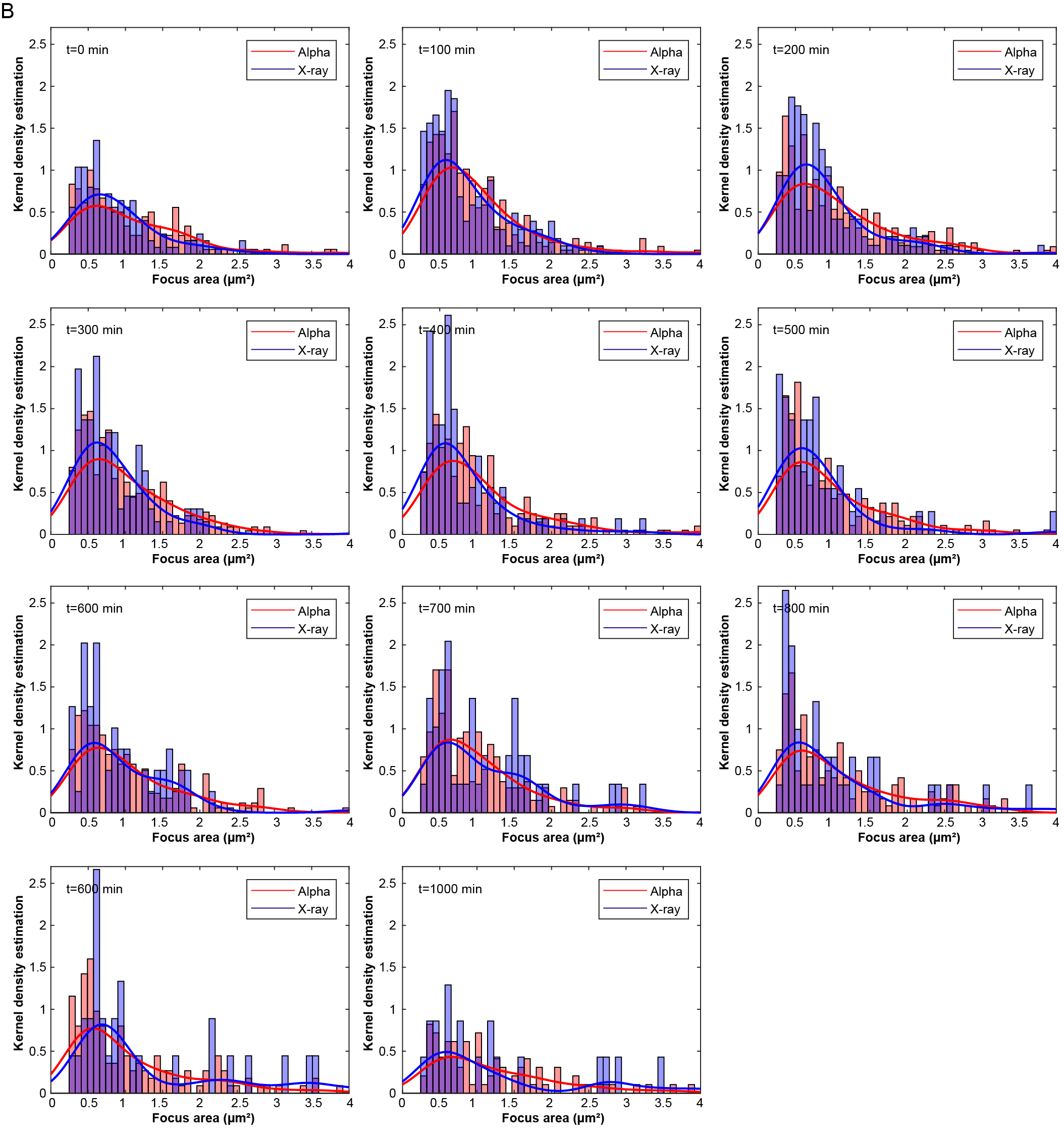

Supplement: Supplementary file 1 [file ijms-21-06602-s001.zip › ijms-884282-supplementary final/Figure S3.jpg]

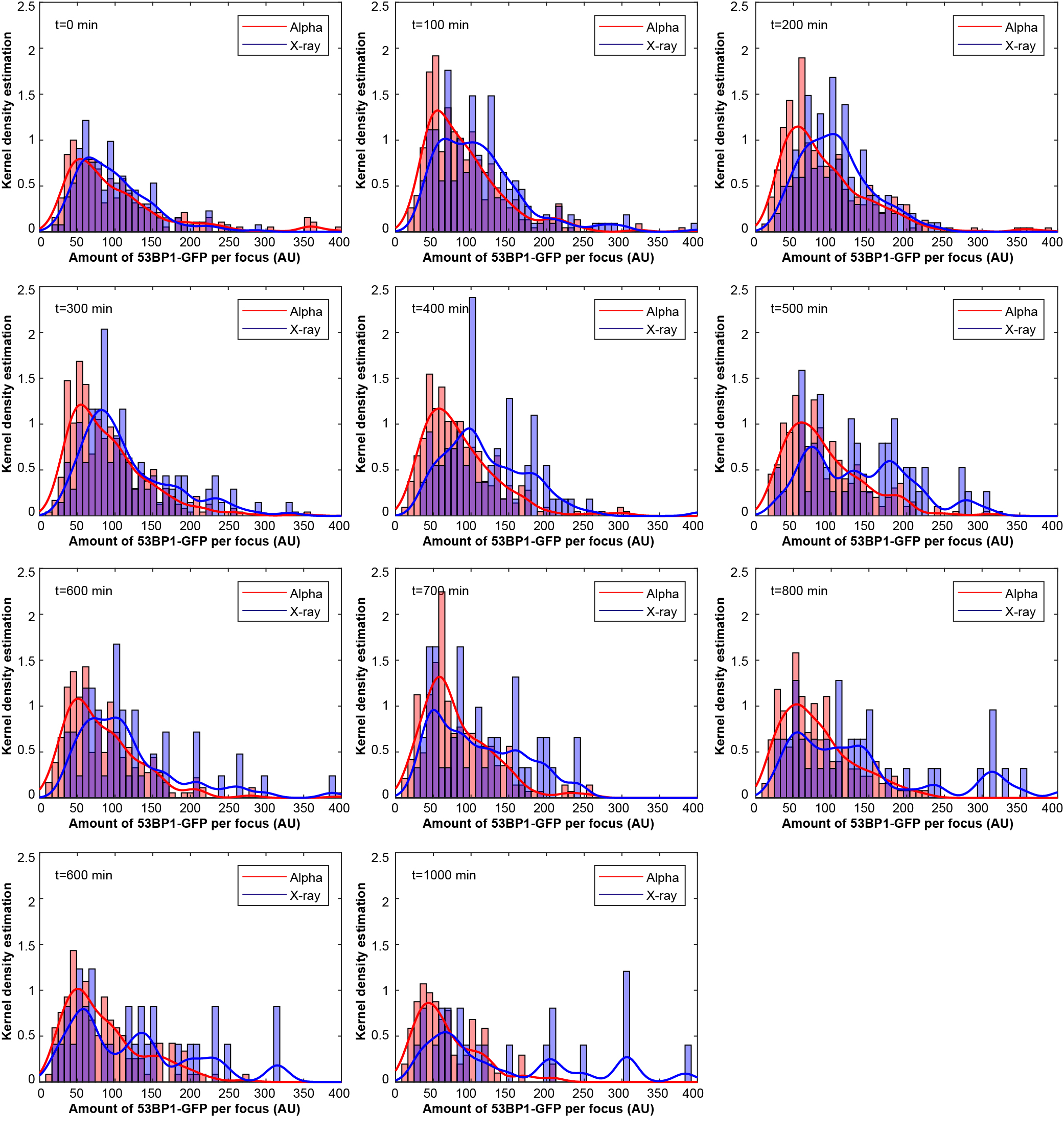

Supplement: Supplementary file 1 [file ijms-21-06602-s001.zip › ijms-884282-supplementary final/Figure S4.jpg]
